# Design of a Differential Capacitive Horizontal Pendulum Tiltmeter

**DOI:** 10.3390/s26144366

**Published:** 2026-07-09

**Authors:** Xiaodong Li, Yinchao Lian, Dongxiao Guan, Jianming Liu, Xinbai Pang, Mengmeng He

**Affiliations:** Earthquake Agency of Xinjiang Uygur Autonomous Region, Urumqi 830011, China; lxdxj12345@163.com (X.L.); guandongxiao3720@163.com (D.G.); inner0991@163.com (J.L.); pxb6610@163.com (X.P.); hmm3161@163.com (M.H.)

**Keywords:** horizontal pendulum, electrostatic feedback, differential capacitive sensor

## Abstract

This paper presents a differential capacitive horizontal pendulum tiltmeter based on electrostatic feedback force. The system mainly consists of a horizontal pendulum bob, a triangular platform, a pendulum locking motor, a differential capacitive sensing circuit, an electrostatic feedback circuit, and a sealed protective housing. The electrostatic feedback differential capacitive horizontal pendulum tiltmeter does not introduce a novel tilt measurement principle; its pendulum still adopts the classic Zöllner double-suspension-wire configuration. In this study, engineering improvements were implemented on the conventional quartz horizontal pendulum tiltmeter by replacing quartz fibers with tungsten wires, substituting optical lever sensors with capacitive sensors, and incorporating the electrostatic feedback principle. The improved tiltmeter features a compact structure and reduced size, which greatly facilitates deployment and installation while significantly enhancing practical applicability. Moreover, it overcomes the drawback of conventional horizontal pendulums—namely, that the scale factor varies with inclination and requires frequent calibration—and thus holds considerable importance for geophysical ground deformation observation.

## 1. Introduction

From the early 1900s to 1930s, geophysicists such as Hugo Benioff developed practical, high-precision horizontal pendulum tiltmeters, which were primarily used to study Earth tides and earthquake precursors. Early horizontal pendulum tiltmeters employed metal suspension wires, offering high mechanical reliability. However, due to the inherent characteristics of the horizontal pendulum system, measurement system sensitivity is inversely related to the torsional stiffness of the suspension wire: lower torque results in a higher mechanical amplification factor and thus greater sensitivity.

With the advancement of quartz materials in the 1970s, quartz suspension wires—characterized by much lower torsional stiffness than metal wires—gradually replaced metal wire, leading to the development of V-M quartz horizontal pendulum tiltmeters with enhanced sensitivity. In the 1990s, the SQ-70D quartz horizontal pendulum tiltmeter was successfully developed. By incorporating a CCD sensor and an optical lever detection system, the instrument achieved digital operation with a tilt resolution better than 0.0002″. It is capable of recording clear solid Earth tides, co-seismic responses, and weak crustal deformations, and has been widely used in geophysical observation networks, becoming an important instrument for deformation monitoring.

**Figure 1:**
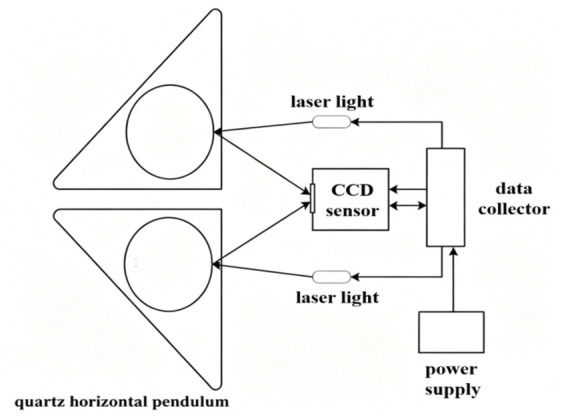
Schematic diagram of the SQ-70D quartz horizontal pendulum.

Figure 1 shows a schematic diagram of the SQ-70D quartz horizontal pendulum. The pendulum adopts the classical Zöllner double-suspension configuration, consisting of suspension wires, a pendulum rod, and a pendulum mass. The pendulum rod is suspended from the frame by two wires and remains nearly horizontal. The sensing system includes a CCD photoelectric converter, a light source, and a dual-channel data acquisition unit. A reflective mirror mounted on the pendulum mass forms an optical lever with the light source, providing a more than tenfold amplification of tilt signals.

The most distinctive feature of the horizontal pendulum tiltmeter is its unique suspension structure, which provides a mechanical amplification factor exceeding three orders of magnitude. However, this amplification factor depends on the tilt angle of the pendulum system. As ground tilt varies during operation, the calibration factor changes accordingly, necessitating frequent recalibration. Furthermore, to achieve high sensitivity, the suspension wire is typically made of quartz with a diameter of approximately 50 μm, making the system highly fragile. The wire is prone to breakage during transportation and installation, and, even during operation, strong seismic events may cause failure. In addition, the optical lever path, typically about 2 m in length, requires a large installation space, limiting the instrument’s practical applicability [1,2,3,4,5].

To improve the reliability and practicality of horizontal pendulum tiltmeters and to eliminate the need for frequent calibration, this study adopts a metal suspension wire to enhance the mechanical strength of the pendulum system. To compensate for the resulting loss in sensitivity, a high-sensitivity parallel three-plate differential capacitive sensor is introduced as the sensing unit. The output voltage signal corresponding to tilt is fed back to the central plate through a feedback circuit, generating an electrostatic feedback force in conjunction with constant DC voltages applied to the outer plates. Through force balance, both the calibration factor and the transfer function of the tiltmeter are stabilized.

The improved CHP (Capacitive Horizontal Pendulum) tiltmeter thus combines enhanced mechanical robustness with high measurement performance. The use of a metal suspension wire significantly increases system reliability, while the differential capacitive sensing circuit compensates for sensitivity loss. The electrostatic feedback mechanism maintains a constant calibration factor, eliminating the need for frequent recalibration. These improvements are of considerable importance for high-precision geophysical monitoring of ground deformation.

## 2. Design of a Differential Capacitive Metal Horizontal Pendulum Tiltmeter Based on Electrostatic Feedback

**Figure 2:**
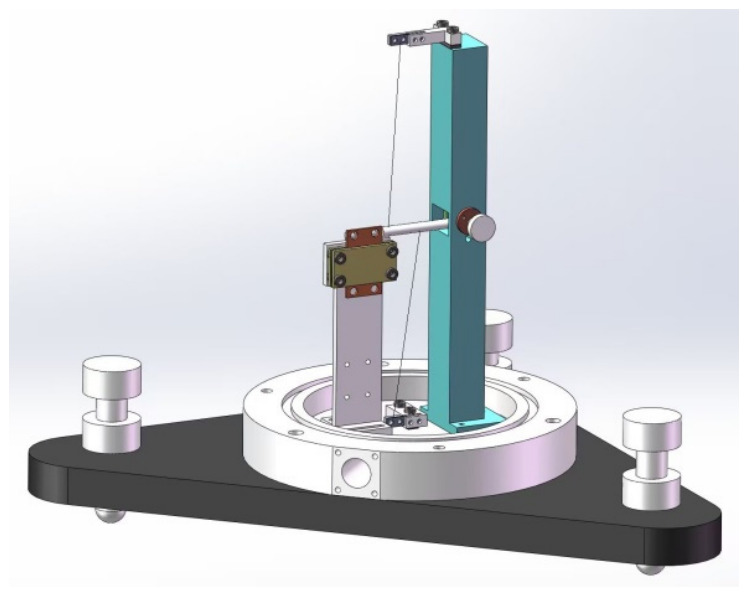
Schematic diagram of the CHP-type capacitive horizontal pendulum tiltmeter based on electrostatic feedback.

The CHP-type capacitive horizontal pendulum tiltmeter based on electrostatic feedback consists of a horizontal pendulum assembly, a triangular platform, a pendulum locking motor, a differential capacitive sensing unit, an electrostatic feedback circuit, and a sealed protective enclosure. As shown in Figure 2, the horizontal pendulum serves as the core sensing element and is mounted on a triangular platform. Driven by a high-precision geared stepper motor, the platform functions both as a leveling mechanism and as a mechanical calibration device.

The pendulum body is equipped with a locking motor that lifts and secures the pendulum rod during transportation or when the instrument is not in operation, ensuring that the suspension wires remain in a relaxed state. Tilt measurements are performed using a differential capacitance measurement system, which includes an excitation source, output transformer, signal preamplifier, AC amplifier, phase-sensitive detector, low-pass filter, and electrostatic feedback circuitry [6,7].

### 2.1. Design of the Horizontal Pendulum Body Assembly

**Figure 3:**
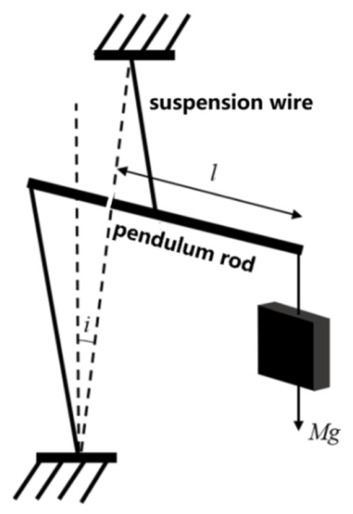
Zöllner double-suspension configuration of the horizontal pendulum.

The horizontal pendulum adopts the classical Zöllner double-suspension configuration. As illustrated in Figure 3, the pendulum system consists of suspension wires, a pendulum rod, and a pendulum mass. The pendulum rod is suspended from the frame by upper and lower wires and remains nearly horizontal. A pendulum mass is attached to one end of the rod and serves as the central plate of the differential capacitive sensor.

In Figure 3, *i* denotes the angle between the rotation axis of the pendulum and the vertical direction, and *l* is the distance from the center of mass of the pendulum to the rotation axis. The measurement direction is perpendicular to the plane defined by the rotation axis and the center of mass. When the ground tilts, the equilibrium position of the pendulum shifts accordingly. If the ground tilts by an angle ψ, the pendulum rod deflects by an angle φ to restore equilibrium. The static equilibrium equation of the system is given by [8,9,10]:

(1) Mgl·sinψ−Mgl·sini·sinφ−ηφ=0

where η denotes the torsion coefficient of the suspension wire, M represents the pendulum mass, and g is the acceleration due to gravity.

Under typical operating conditions, i, ψ, and φ are all small angles; thus, sini≈i, sinψ≈ψ, and sinφ≈φ. The above equation can therefore be simplified to:

(2) $$\frac{\varphi }{\psi }=\frac{Mgl}{Mgli+\eta }=\frac{1}{i+\frac{\eta }{Mgl}}=\frac{1}{i+\varepsilon }$$

where $\left(\varepsilon =\frac{\eta }{Mgl}\right)$.

Equation (2) defines the ratio of pendulum deflection to ground tilt, i.e., the mechanical amplification factor of the horizontal pendulum. This factor depends on both the deviation angle *i* and the torsion coefficient of the suspension wire. In this design, a tungsten suspension wire with a diameter of 100 μm is used, resulting in a small torsion coefficient. Since *i* is also very small under normal conditions, (typically within 5″), the system achieves a large amplification factor, generally on the order of several thousand.

However, Equation (2) also indicates that the amplification factor depends on *i*, which varies with ground tilt. Therefore, the calibration factor of conventional horizontal pendulum tiltmeters is not constant and requires frequent recalibration during operation.

Equation (2) further shows that the torsion coefficient of the suspension wire has a significant influence on measurement sensitivity: the smaller the torsion coefficient, the higher the amplification factor and sensitivity. Early horizontal pendulum tiltmeters employed tungsten wires; however, quartz wires, with much lower torsional stiffness, were later adopted to achieve higher sensitivity.

With the widespread application of quartz-based instruments, several limitations have become evident. Although quartz wires have high tensile strength, they are significantly more brittle than tungsten wires and are prone to fractures during transportation, installation, or strong seismic events. Furthermore, quartz wires require fusion bonding to the pendulum rod and frame, making fabrication and installation complex and costly. Consequently, improving the robustness and reliability of suspension wires has become a key challenge.

In the proposed CHP tiltmeter, tungsten wire is used as the suspension material due to its superior toughness, significantly enhancing mechanical reliability. However, this increases the torsion coefficient and reduces the mechanical amplification factor. For a uniform circular filament of diameter d and length L, the torsional coefficient η is given by $\eta =\frac{GJ_{p}}{L}$, where G is the shear modulus of the material and $J_{p}=\frac{\pi d^{4}}{32}$ is the polar moment of inertia of the circular cross-section. This yields the explicit expression $\eta =\frac{\pi Gd^{4}}{32L}$. Taking the shear modulus as 3.0 × 10^10^ Pa for fused quartz and 16.0 × 10^10^ Pa for tungsten, we note that conventional quartz horizontal pendulums typically employ quartz fibers with a diameter of 50 μm, whereas the CHP-type capacitive horizontal pendulum tiltmeter adopts a tungsten wire with a diameter of 100 μm. A simple estimation indicates that the torsion coefficient of the tungsten suspension in the CHP instrument is approximately 85 times greater than that of the quartz fiber. Consequently, the mechanical advantage of the system is substantially reduced.

To compensate for this limitation, a high-precision differential capacitive sensing system is employed, combined with bridge measurement, electronic amplification, and phase-sensitive detection. These electronic components provide signal amplification by several orders magnitude, effectively offsetting the loss in mechanical sensitivity.

Moreover, compared with the optical lever system used in quartz tiltmeters, the capacitive sensing approach requires less space, enabling a more compact design and improved system integration.

As shown in Figure 2, the pendulum rod, mass, and suspension wires are all made of metal. Two fixed metal plates are positioned on either side of the pendulum mass, forming a three-plate differential capacitor with an effective gap of approximately 0.3 mm (excluding the central plate). The pendulum mass acts as the movable central electrode. When ground tilt causes the pendulum to deflect, the central plate is displaced, resulting in change in capacitance.

As a highly sensitive displacement sensor, the differential capacitive system can achieve a displacement resolution on the order of 10^−11^ m. By leveraging this high sensitivity, the proposed design simultaneously satisfies the requirements for improved reliability and high measurement precision [11].

### 2.2. Design of the Differential Capacitive Ground Tilt Circuit Based on Electrostatic Feedback

**Figure 4:**
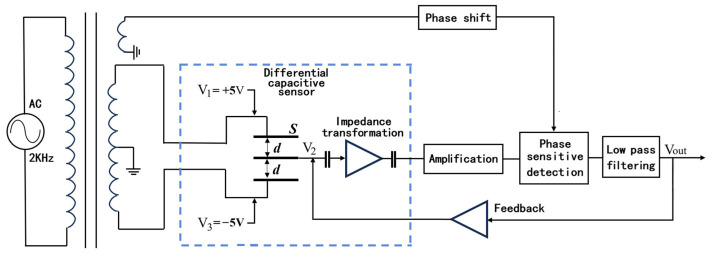
Differential capacitive ground tilt circuit based on electrostatic feedback.

As shown in Figure 4, two capacitor plates are arranged on either side of the metal horizontal pendulum mass, forming a three-plate differential capacitor in which the pendulum mass serves as the central electrode. An AC excitation signal is applied to the two side plates through a center-tapped output transformer. Together, the transformer and the differential capacitor constitute a measurement bridge.

The unbalanced bridge signal is processed sequentially through a preamplifier, AC amplifier, phase-sensitive detector, and low-pass filter. Meanwhile, DC voltages of equal magnitude and opposite polarity are applied to the two side plates. The output voltage of the measurement bridge is fed back to the central plate via a feedback circuit, generating an electrostatic force that restores the central plate to its equilibrium position. In this way, an electrostatic force negative feedback system is established [11,12,13,14].

In this configuration, the DC voltages applied to the side plates generate the electrostatic force, while the output signal $V_{out}$ represents the displacement of the pendulum mass from its central position. This signal is fed back to the central plate, and under the action of the applied DC voltages, an electrostatic feedback force is produced, maintaining the pendulum mass in a force-balanced state.

The electrostatic force between parallel plates can be derived by neglecting edge effects and assuming a uniform electric field perpendicular to the plates. If the voltage across the two plates is U, the electric field is $E=\frac{U}{d}$, where d is the plate spacing. The charge on the plate is $Q=CU=\frac{\varepsilon_{0}SU}{d}$, where $\varepsilon_{0}$ is the permittivity of free space and S is the plate area. The electrostatic force acting on one plate due to the other is then: $F_{q}=\frac{1}{2}EQ=\frac{\varepsilon_{0}SU^{2}}{{2d}^{2}}$.

As shown in Figure 4, for a three-plate capacitive structure, DC bias voltages $V_{1}$ and $V_{3}$ are applied to the top and bottom plates, respectively, with the potential difference between them defined as $=V_{1}-V_{3}$. A DC feedback voltage $V_{2}$ is applied to the middle plate, and the net electrostatic force acting on the central plate is:

(3) $$F=\frac{\varepsilon_{0}S{\left(V_{1}-V_{2}\right)}^{2}}{2d^{2}}-\frac{\varepsilon_{0}S{\left(V_{3}-V_{2}\right)}^{2}}{2d^{2}}$$

The DC bias voltages $V_{1}\;$and $V_{3}$ applied to the two outer plates are equal in magnitude and opposite in polarity; the force simplifies to:

(4) $$F=\frac{\varepsilon_{0}S{\left(V_{1}-V_{2}\right)}^{2}}{2d^{2}}-\frac{\varepsilon_{0}S{\left(V_{3}-V_{2}\right)}^{2}}{2d^{2}}=\frac{2\varepsilon_{0}SUV_{2}}{d^{2}}$$

According to industry standards, a high-precision underground cavern tiltmeter should achieve a resolution better than 0.0002″, with a measurement range of at least 2″. This corresponds to a pendulum acceleration range of approximately $g\sin2\prime \prime \approx 10^{-4}\;m/s^{2}$. The proof mass of the CHP-type horizontal pendulum tiltmeter is approximately 10 g. According to Newton’s second law (F=Ma), the maximum feedback force required by the system is on the order of 10^−6^ N.

The effective gap spacing between the outer plates of the CHP-type horizontal pendulum tiltmeter’s capacitive sensor is approximately 0.3 mm, and the plate area is approximately 1000 mm^2^. DC bias voltages of +5 V and −5 V are applied to the opposing capacitor plates, resulting in a 10 V potential difference across the electrode pair. The feedback voltage applied to the central plate can reach $V_{2}=\pm 5\;V$, producing a maximum electrostatic force of approximately 10^−5^ N, which is sufficient to meet system requirements.

### 2.3. Calibration of the Horizontal Pendulum Tiltmeter Based on Electrostatic Feedback

The derivations presented in this chapter are drawn from references [8,9,10]. The horizontal pendulum can be regarded as an instrument for measuring the horizontal component of acceleration. When the ground tilt angle is ψ, the corresponding horizontal acceleration is gsinψ. By measuring this component, the tilt angle can be determined. Therefore, the horizontal pendulum system can be analyzed using the theory of pendulous accelerometers.

Let the input acceleration be $x_{a}$. The equation of motion of the pendulum is given by [8,10]:

(5) $$J\frac{d^{2}\varphi }{dt^{2}}+F_{D}l^{2}\frac{d_{\varphi }}{dt}+\left(Mgl\sin i+\eta \right)\varphi =Mlx_{a}$$

where J is the moment of inertia (for this system, $J=Ml^{2}$) and $F_{D}$ is the damping coefficient. By letting y=lφ represent the displacement of the pendulum mass, the equation becomes:

(6) $$Ml\frac{d^{2}y}{dt^{2}}+F_{D}l\frac{dy}{dt}+\left(Mg\sin i+\frac{\eta }{l}\right)y=Mlx_{a}$$

Applying the Laplace transform yields the transfer function:

(7) $$H\left(s\right)=\frac{Y(s)}{X_{a}(s)}=\frac{1}{s^{2}+\frac{F_{D}}{M}s+\frac{g\sin i}{l}+\frac{\eta }{Ml^{2}}}$$

**Figure 5:**
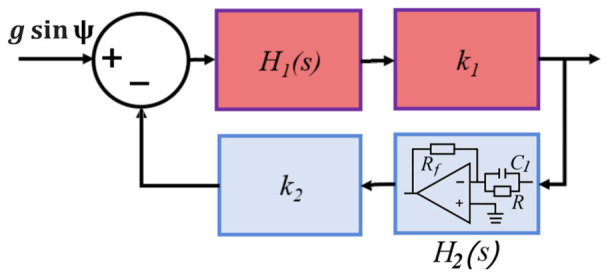
Transfer function of the horizontal pendulum tiltmeter based on electrostatic feedback.

In the system shown in Figure 5, $H_{2}\left(s\right)$ represents the transfer function of the mechanical pendulum system and can be expressed as Equation (7). The differential capacitive circuit converts displacement into voltage with sensitivity $k_{1}$. The feedback circuit is implemented using an inverting proportional amplifier and can be regarded as comprising two parallel feedback paths: a linear path and a derivative path, formed by resistors and capacitors. Its transfer function can be expressed as:

(8) $$H_{2}\left(s\right)=-{R_{f}C}_{1}s-\frac{R_{f}}{R}$$

Let p and q denote the derivative and proportional transmission coefficients of the feedback network, respectively, where $p={-R_{f}C}_{1}$ and $=-\frac{R_{f}}{R}$.

(9) $$H_{2}\left(s\right)=ps+q$$

The feedback actuator converts voltage to force with coefficient $k_{2}$ (N/V). The overall transfer function of the electrostatic feedback tiltmeter is:

(10) $$H\left(s\right)=\frac{H_{1}(s)k_{1}}{1+\frac{H_{1}(s)k_{1}H_{2}(s)k_{2}}{M}}=\frac{k_{1}}{s^{2}+\frac{F_{D}+k_{1}k_{2}p}{M}s+\frac{k_{1}k_{2}ql^{2}+Mgl\sin i+\eta }{Ml^{2}}}$$

This represents a second-order low-pass system describing the response of output voltage to input acceleration. The sensitivity of the output voltage to acceleration is:

(11) $$S_{a}=\frac{k_{1}Ml^{2}}{k_{1}k_{2}ql^{2}+Mgl\sin i+\eta }\approx \frac{M}{k_{2}q}$$

Since i and η are very small and $k_{1}$ is relatively large (indicating strong feedback), the sensitivity can be approximated as $\frac{M}{k_{2}q}$.

These results show that, in the electrostatic feedback differential capacitive system, the sensitivity depends only on the pendulum mass *M*, the feedback force coefficient $k_{2}$, and the proportional feedback coefficient *q*, and is independent of the suspension parameters (angle *i*) and the torsion coefficient (η).

Because *M*, $k_{2}$, and *q* are constants, the calibration factor of the tiltmeter remains constant. This eliminates the need for frequent calibration or adjustment, representing a key advantage over conventional horizontal pendulum tiltmeters.

### 2.4. Design of the Triangular Platform and Pendulum Locking Mechanism

The structural design of the horizontal pendulum tiltmeter inherently limits its dynamic range. In practical applications, the pendulum mass must be balanced such that it remains at the central position between the capacitor plates. Therefore, horizontal pendulum tiltmeters are typically installed on an adjustable tilt platform. The triangular platform used in this system is illustrated in Figure 2. It is designed as an isosceles right triangle, with the right-angle vertex serving as a fixed support foot, while adjustable support feet are installed beneath the two equal sides to enable leveling adjustments.

Each adjustable foot is driven by a high-precision geared stepper motor and controlled programmatically, allowing tilt adjustment within a range of ±15°. Given that the positioning accuracy of the stepper motor can reach 1 μm, the adjustable platform also functions as a calibration mechanism for the pendulum system, enabling in situ calibration of linearity and sensitivity over a wide range.

The pendulum system is the most fragile component of the tiltmeter. To enhance the instrument’s stability and impact resistance, a pendulum locking mechanism is installed on the pendulum rod. During transportation or installation, this mechanism clamps one end of the pendulum rod and lifts it slightly, thereby keeping both suspension wires in a stress-free state. In addition, the pendulum system and the upper platform are enclosed within a sealed protective housing, which provides waterproofing and moisture resistance, ensuring reliable operation under harsh environmental conditions.

Compared with quartz suspension, metal suspension wires exhibit significantly greater strength and toughness. Consequently, when the pendulum is locked and appropriate external packaging is applied, the instrument can withstand routine transportation and even drop impacts of up to 1 m, demonstrating high reliability [15,16,17,18,19].

## 3. Calibration Tests of the CHP Capacitive Horizontal Pendulum Tiltmeter

The CHP-type capacitive horizontal pendulum tiltmeter presented in this paper represents an engineering improvement over conventional horizontal pendulum tiltmeters. By replacing the optical lever with a differential capacitive sensing unit, the instrument not only reduces its overall size and achieves a more compact structure, but also overcomes the dependence of the scale value on the i angle, which is a well-known limitation of traditional instruments.

To verify these improvements, calibration experiments were conducted on a high-precision tilt platform capable of providing accurate tilts in two orthogonal directions. During testing, the platform was tilted stepwise at 1″ increments along the measurement direction of the pendulum to record the instrument output. From these data, the sensitivity and linearity of the tiltmeter were obtained. Subsequently, the platform was tilted at 3″ increments along the i angle direction, and the device was recalibrated. By comparing the sensitivity and linearity obtained from multiple calibrations under different i angles, it is possible to confirm whether the proposed capacitive horizontal pendulum tiltmeter eliminates the undesirable variation in the scale value with the *i*-angle.

Since the i angle of a horizontal pendulum is very small and difficult to measure directly, it is conventionally characterized by the natural oscillation period of the pendulum. In the initial calibration, with the electrostatic feedback disabled, the measured natural period was 40 s; the corresponding i angle is denoted as$\;i_{0}$.

Calibration results under different *i*-angles show that the CHP capacitive horizontal pendulum tiltmeter has a measurement range exceeding 6″. The sensitivities are 575.35 mV/″ (Table 1), 574.96 mV/″ (Table 2), and 574.81 mV/″ (Table 3), respectively, while the linearity values are 0.26%, 0.72%, and 0.77%. The maximum deviation in sensitivity is less than 1%, demonstrating that the scale value remains essentially unchanged across different i angle conditions.

## 4. Field Observations of the CHP-Type Capacitive Horizontal Pendulum Tiltmeter

Field observations of the CHP-type capacitive horizontal pendulum tiltmeter were conducted in October 2025. The observation station was located in an underground cavern at the Chongli Seismic Station in Zhangjiakou, Hebei Province. The cavern has a depth of 70 m, with an overburden thickness exceeding 30 m in the observation area. The annual average temperature variation is within 1 °C, providing a stable environment for high-precision measurements.

The tiltmeter was installed in a side chamber located approximately 60 m from the entrance, where a dedicated seismic monitoring pier is available. Shortly after installation, the instrument successfully recorded clear solid Earth tides.

To meet the observational requirements of the China National Seismic Network (CNSN) and capture clear solid Earth tide signals, the output of the ground tiltmeter must be processed with a high-order low-pass filter to suppress microseismic noise. The filter cutoff frequency is set at no higher than 0.0082 Hz (corresponding to a 120 s period). Consequently, the effective observation band can be regarded as quasi-DC, and the instrument sampling rate is set to 1 min.

### 4.1. Venedikov Harmonic Analysis of Tilt Observation Data

**Figure 6:**
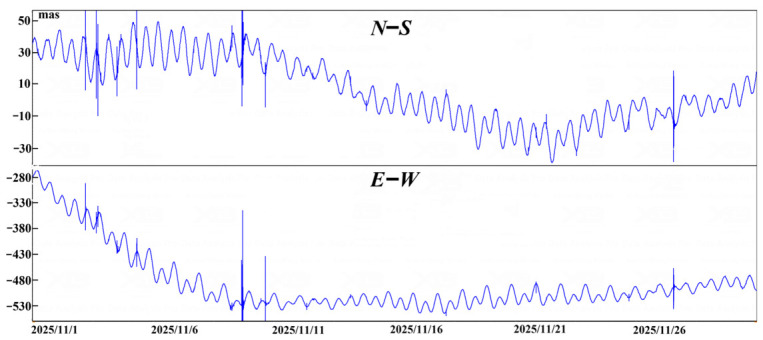
Observation data curve of the CHP capacitive horizontal pendulum tiltmeter in November 2025.

Shortly after installation, the tiltmeter recorded clear Earth tides and co-seismic responses. Figure 6 shows the observation curve for November 2025, where spring and neap tides are distinctly visible, and multiple seismic co-seismic signals are recorded.

According to the Technical Standard for Seismic Observation Instruments Entering the Network (DB/T 31.1–2008) [20], the mean square error of the M_2_ wave derived from Venedikov harmonic analysis must be less than 0.02 to meet the observation specification. Table 4 presents the results of the harmonic analysis for the CHP capacitive horizontal pendulum tiltmeter deployed in the Chongli cave during November 2025. The mean square errors of the M_2_ wave in the north–south and east–west components are 0.0106 and 0.0058, respectively, both satisfying the regulatory requirements.

### 4.2. Co-Seismic Responses Recorded by the CHP Capacitive Horizontal Pendulum Tiltmeter

**Figure 7:**
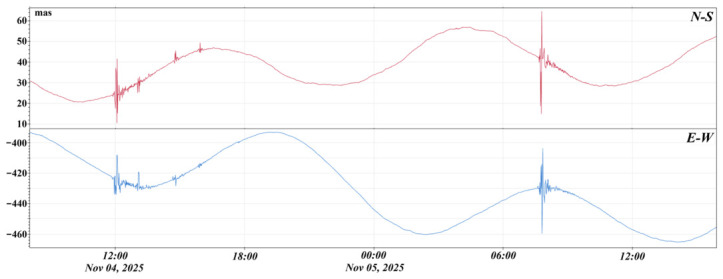
Co-seismic signals recorded by the tiltmeter between 4 and 5 November 2025.

Figure 7 shows the co-seismic signals recorded between 4 and 5 November 2025. Table 5 lists global earthquakes with magnitudes ≥ M5.0 during the same period. Data are sourced from: https://earthquake.usgs.gov/earthquakes/map/ (accessed on 1 July 2026).

According to the observation specifications of the China Earthquake Networks Center (CENC), in order to resolve clear solid Earth tides, the tiltmeter output must be processed with a high-order low-pass filter to suppress microseismic noise. The filter cutoff frequency is set no higher than 0.0082 Hz (corresponding to a period of 120 s). Consequently, the effective observation band is effectively quasi-DC, with a sampling interval of 1 min. Therefore, any co-seismic responses are utilized only as a reference for evaluating the operational status of the instrument.

### 4.3. Estimation of the Resolution of the CHP Capacitive Horizontal Pendulum Tiltmeter Based on Solid Earth Tides

According to the Technical Standard for Seismic Observation Instruments Entering the Network (DB/T 31.1–2008), the resolution of a horizontal pendulum tiltmeter must reach 0.0002″. Since existing metrological equipment make it difficult to carry out such detection and calibration under laboratory conditions, the resolution is generally inferred from solid Earth tides in geodesy and seismological studies.

Theoretical solid tides are calculated using a Homogeneous Elastic Sphere model, introducing the Love–Shida numbers (h, k, l). The Earth is assumed to be a uniform, isotropic, perfectly elastic sphere. The calculation methodology references Appendix A of DB/T 45-2012 The Method of Earthquake-Related Crustal Deformation Monitoring—Crustal Tilt Observation [21].

**Figure 8:**
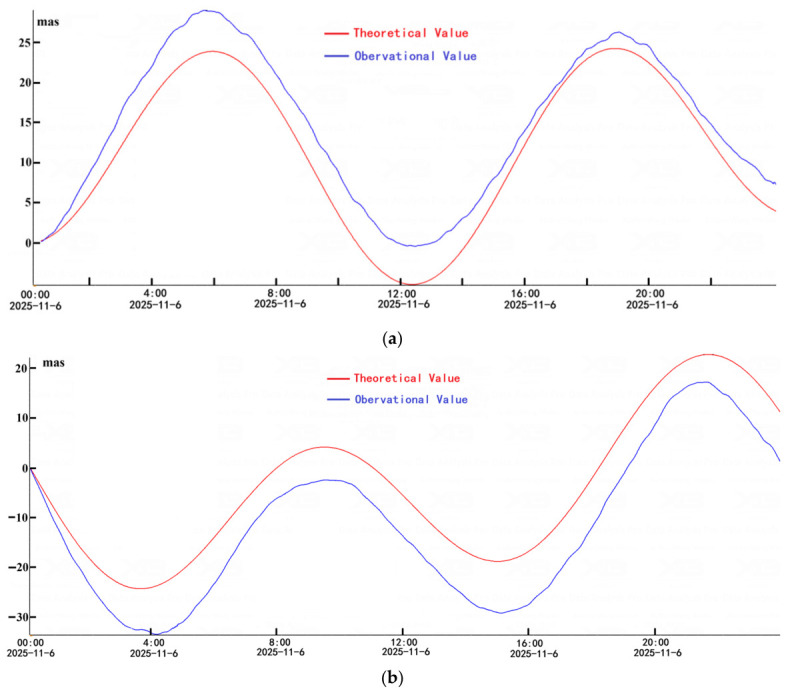
(**a**). Theoretical and observed north–south tilt earth tide curves at the Chongli Station, Zhangjiakou, on 6 November 2025. (**b**). Theoretical and observed east–west tilt earth tide curves at the Chongli Station, Zhangjiakou, on 6 November 2025.

Taking the spring-tide observation data (6 November 2025) as an example, the observed values and the theoretical solid-tide curve were plotted in the same coordinate system, with both curves shifted so that their initial points coincided at zero. As shown in Figure 8a,b, the observed and theoretical curves exhibit similar waveforms with only a small phase deviation.

According to Annex B of the Technical Standard for Seismic Observation Instruments (DB/T 31.1–2008), the resolution of the measurement system is estimated by comparing the observed tilt data with the theoretical solid Earth tide.

Select a relatively smooth time period from the observed curve during the spring tide phase and calculate the theoretical values of the solid-tide corresponding to this period. Identify the peak (maximum value point) in the theoretical solid tide value series, denoting it as $d_{0}$, and the corresponding time as $t_{0}$. Using this point as the center, select 2n points (where n is the number of points taken from one side) on either side where the inclination value changes by ∆d. Record the corresponding theoretical values and times, denoting them as $d_{i}$ and $t_{i}$, respectively. The change in inclination ∆d should be chosen within the range of 0.0001″/2.0 min to 0.0001″/2.5 min.

Based on the selected time period in the theoretical solid-tide value series, locate the positions of the peaks in the observed data series within the corresponding time period. According to the time intervals ($T_{i}$) of the selected points in the theoretical solid-tide series, find the corresponding data points on both sides of the observed data peaks, denoting them as $d_{i}^{\prime }$. Substitute $d_{i}^{\prime }$ into Formula (12) to calculate the normalization coefficient, denoted as k. Multiply the selected points $d_{i}^{\prime }$ from the observed data series by the normalization coefficient k to obtain the normalized series, denoted as $d_{i}^{\prime }$. Substitute $d_{i}^{\prime \prime }$ into Formula (13) to obtain the fitted series, denoted as $\bar{d_{i}^{\prime \prime }}$. Calculate the difference between the normalized series $d_{i}^{\prime \prime }$ and the fitted series $\bar{d_{i}^{\prime \prime }}$ to obtain the difference series $d_{i}^{\prime \prime }$. Lastly, select the maximum value between $∆d_{-2}^{\prime \prime }$ and $∆d_{2}^{\prime \prime }$; this value represents the instrument’s resolution.

(12) $$k=\frac{2\times (n-2)}{\left(d_{n}^{\prime }-d_{2}^{\prime }\right)+(d_{-n}^{\prime }-d_{-2}^{\prime })}\times \frac{0.0001\prime \prime }{0.001\prime \prime }$$

(13) $$\bar{d_{i}^{\prime \prime }}=(\frac{\sum_{i=2}^{n}d_{i}^{\prime \prime }}{\left(n-2\right)+1}+\frac{\sum_{i=-2}^{-n}d_{i}^{\prime \prime }}{\left(n-2\right)+1})/2$$

According to the specification, operators may select the optimal peaks and troughs from the observation data; a resolution better than 0.0002″ is considered to meet the standard requirement.

**Figure 9:**
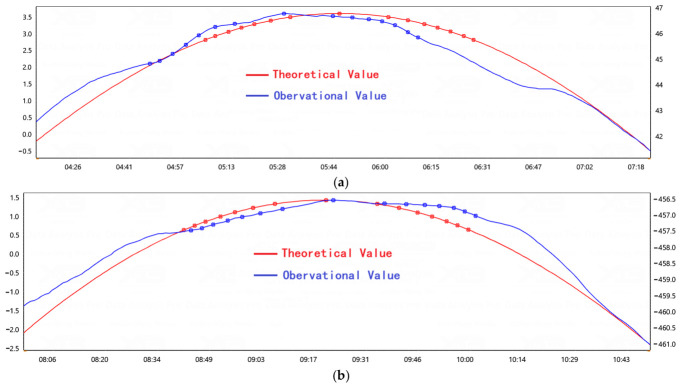
(**a**). Resolution curve of the north–south tilt component at the Chongli Station estimated from theoretical solid Earth tides. (**b**). Resolution curve of the east–west tilt component at the Chongli Station estimated from theoretical solid Earth tides.

Based on the resolution estimation from solid Earth tides presented in Table 6 and Table 7 (calculated using the data points selected in Figure 9a,b), the CHP capacitive horizontal pendulum tiltmeter achieves a resolution of 0.1737 × 10^−3^″ in the north–south component and 0.1904 × 10^−3^″ in the east–west component. Both values are significantly better than the required 0.0002″, thus meeting the design specifications.

### 4.4. Power Spectral Density Analysis of Observation Data from the CHP-Type Capacitive Horizontal Pendulum Tiltmeter

The power spectral density (PSD) was used to analyze the actual observation data of the CHP-type capacitive horizontal pendulum tiltmeter. First, the observation data from November 2025 were detrended to eliminate instrumental zero drift and long-period non-solid-tide drifts. Then, according to the observation principle of the earth tiltmeter, the observed angular values were converted into the tilt components of gravitational acceleration. The conversion formula is: $g_{\varphi }=g\times \tan^{-1}\left(\frac{\varphi }{3600\times 1000}\right)$, where g is the gravitational acceleration, φ is the observed angular value (mas), and $g_{\varphi }$ is the component of gravitational acceleration in the tilt direction.

The Welch averaged periodogram method was adopted to calculate the power spectral density of the acceleration sequence. The window length was set to 4096 points (approximately 68.3 h), which can cover the frequencies of the main solid Earth tide families. The overlap ratio was 50% (2048 points). The number of FFT points was twice the window length. A Hanning window was selected for its balanced main lobe and side lobe characteristics, making it suitable for continuous vibration signals.

**Figure 10:**
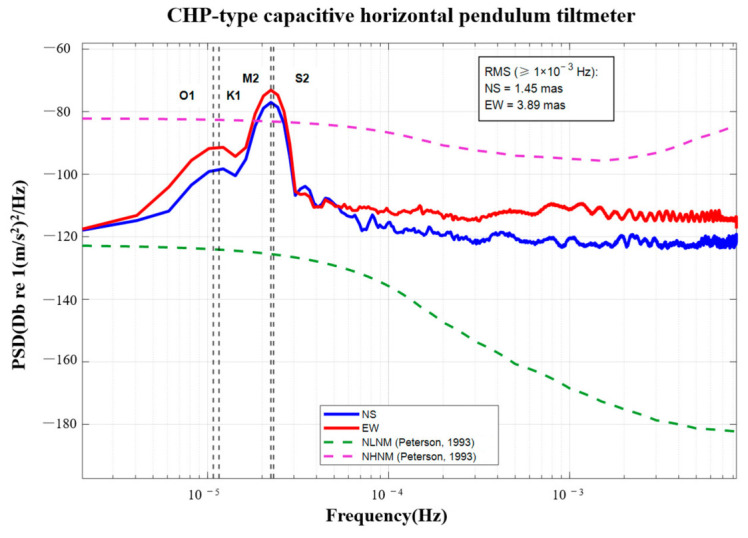
Power spectral density (PSD) of the observation data from the CHP-type capacitive horizontal pendulum tiltmeter for November 2025 [22].

Figure 10 shows the acceleration power spectral density (PSD) curve of the CHP-type capacitive horizontal pendulum tiltmeter for November 2025. The frequencies of the major tidal wave groups O_1_, K_1_, M_2_, and S_2_ are marked to identify deterministic geophysical signals within the observational data. The global seismic background noise models of Peterson (1993) [22], namely the New Low Noise Model (NLNM) and the New High Noise Model (NHNM), are included as reference baselines for station noise levels. Overall, except for the solid Earth tide band, the observed noise lies between the NLNM and NHNM curves, consistent with the general characteristics of global seismic background noise.

It is generally accepted that low-frequency signals in ground tilt observations consist of solid Earth tides, linear drift, and crustal deformation, whereas significant noise appears above 10^−3^ Hz. This higher-frequency noise includes microseisms, co-seismic responses, environmental disturbances, and instrumental electrical noise. Consequently, the RMS noise of the CHP-type capacitive horizontal pendulum tiltmeter was calculated by integrating the PSD curve over the high-frequency band (f ≥ 10^−3^ Hz) using the trapezoidal rule. The resulting RMS noise levels are 1.45 mas for the NS component and 3.89 mas for the EW component.

Since the noise above 10^−3^ Hz is a mixture of microseisms, co-seismic responses, and environmental interference, the calculated RMS noise is approximately one order of magnitude higher than that estimated using the conventional solid-tide resolution inference method.

## 5. Discussion

This study presents the design and performance of a CHP-type capacitive horizontal pendulum tiltmeter. By adopting a metal suspension wire, the mechanical strength and reliability of the pendulum system are significantly improved. In addition, the integration of a pendulum locking mechanism, an adjustable tilt platform, and a sealed protective enclosure further enhances the instrument’s robustness and environmental adaptability.

A high-precision differential capacitive sensing circuit is used to compensate for the reduced sensitivity associated with the use of metal suspension wires. Furthermore, the implementation of an electrostatic feedback force balancing technique ensures a constant calibration value, thereby eliminating the need for frequent recalibration.

Field observations demonstrate that the instrument achieves a ground tilt measurement resolution better than 0.0002″ and is capable of clearly recording solid Earth tides and co-seismic responses. It also supports large-range linear calibration and exhibits high reliability, strong environmental adaptability, and operational convenience.

Overall, the proposed tiltmeter provides a robust and practical solution for high-precision geophysical ground deformation monitoring.

Table 8 presents a comparative overview of the technical specifications of the CHP capacitive horizontal pendulum tiltmeter and the SQ-70D quartz horizontal pendulum tiltmeter. It clearly demonstrates the performance advantages of the CHP tiltmeter, particularly in terms of stability and sensitivity.

## Figures and Tables

**Table 1:** Calibration data under the condition of $i_{0}$.

Tilt Amount (″)	Sensor Output (mv)	Fitted Value (mv)	Linear Deviation (mv)
0	−1690.266	−1686	−4.266
1.023	−1095.62	−1097.41695	1.79695
2.067	−499.495	−496.75155	−2.74345
2.994	45.741	36.5979	9.1431
3.987	610.348	607.92045	2.42755
5.001	1185.839	1191.32535	−5.48635
6.012	1771.975	1773.0042	−1.0292
Sensitivity 575.35 mV/″		Linearity 0.26%	

**Table 2:** Calibration data under the condition of $i_{0}$ + 3″.

Tilt Amount (″)	Sensor Output (mv)	Fitted Value (mv)	Linear Deviation (mv)
0	−1752.844	−1727.4	−25.444
0.939	−1169.56	−1187.51256	17.95256
1.973	−579.322	−593.00392	13.68192
3.001	11.991	−1.94504	13.93604
4.072	602.87	613.83712	−10.96712
5.103	1192.554	1206.62088	−14.06688
6.1	1785.036	1779.856	5.18
Sensitivity 574.96 mV/″		Linearity 0.72%	

**Table 3:** Calibration data under the condition of $i_{0}$ + 6″.

Tilt Amount (″)	Sensor Output (mv)	Fitted Value (mv)	Linear Deviation (mv)
0	−1701.223	−1686.2	−15.023
1.011	−1117.749	−1105.06709	−12.68191
2.003	−528.967	−534.85557	5.88857
2.991	60.619	33.05671	27.56229
4.025	650.804	627.41025	23.39375
5.101	1240.23	1245.90581	−5.67581
6.211	1860.186	1883.94491	−23.75891
Sensitivity 574.81 mv/″		Linearity 0.77%	

**Table 4:** Venedikov harmonic analysis of the tiltmeter observation data for November 2025.

Strain Channel	Tidal Factor	Tidal Factor Mean Squared Error	Tidal Phase Lag	Tidal Phase Lag Mean Squared Error
N-S	0.9049	0.0106	5.7154	0.6049
E-W	1.0050	0.0058	−8.0010	0.3331

**Table 5:** Global earthquakes with M ≥ 5.0, 4–5 November 2025.

Origin Time (UTC + 8)	Location		Magnitude (M)	Depth (km)	
4 November 2025 11:39	160.294° E	52.328° N	5.1	17.5	139 km SE of Petropavlovsk-Kamchatsky, Russia
4 November 2025 11:45	160.406°	52.174°	5.9	20	M 5.9–155 km SE of Petropavlovsk-Kamchatsky, Russia
4 November 2025 11:58	160.246°	52.247°	5.3	35	142 km SE of Petropavlovsk-Kamchatsky, Russia
4 November 2025 12:45	160.296°	52.377°	5.5	10	136 km SE of Petropavlovsk-Kamchatsky, Russia
4 November 2025 12:47	159.074°	52.789°	5.1	104.4	42 km SE of Petropavlovsk-Kamchatsky, Russia
4 November 2025 13:13	148.884°	5.809°	5.0	123.4	85 km WNW of Kandrian, Papua New Guinea
4 November 2025 13:14	160.222° E	51.560° N	5.0	10.0	196 km SE of Vilyuchinsk, Russia
4 November 2025 13:29	160.464° E	52.288° N	5.0	10.0	151 km SE of Petropavlovsk-Kamchatsky, Russia
4 November 2025 14:28	160.414° E	52.215° N	5.4	10.0	153 km SE of Petropavlovsk-Kamchatsky, Russia
4 November 2025 15:37	143.143° E	12.392° N	6.0	65.0	191 km WSW of Merizo Village, Guam
5 November 2025 07:28	160.190° E	52.112° N	6.0	28.0	149 km SE of Petropavlovsk-Kamchatsky, Russia
5 November 2025 07:32	123.065° E	0.125° S	5.9	114.6	73 km S of Gorontalo, Indonesia
5 November 2025 07:49	155.857° E	49.725° N	5.7	71.0	107 km S of Severo-Kuril’sk, Russia
5 November 2025 08:33	105.973° W	35.243° S	5.1	10.0	Southern East Pacific Rise

**Table 6:** Resolution estimates of the north–south tilt component at the Chongli Station based on theoretical solid Earth tides.

N	Date	Theory Value	Time Interval	Obs Valus	Norm Value	Fit Value	Difference
	6 November 2025	$d_{i}$	$t_{i}$	$d_{i}^{\prime }$	$d_{i}^{\prime \prime }$	$\bar{d_{i}^{\prime \prime }}$	$∆d_{i}^{\prime \prime }$
	Time	0.001″	min	0.001″	0.001″	0.001″	0.001″
−7	05:05	2.8084	41	44.8180	20.2796	20.4590	0.1793
−6	05:08	2.9176	38	44.9200	20.3258	20.5785	0.2527
−5	05:12	3.0509	34	45.1960	20.4507	20.6980	0.2473
−4	05:16	3.1699	30	45.5580	20.6145	20.8175	0.2030
−3	05:20	3.2746	26	45.9260	20.7810	20.9370	0.1560
−2	05:25	3.3848	21	46.2530	20.9290	21.0565	0.1275
−1	05:31	3.4867	15	46.3660	20.9801	21.1760	0.1959
0	05:46	3.5944	0	46.7720	21.1638	21.2955	0.1317
1	06:01	3.4897	15	46.6660	21.1158	21.1760	0.0601
2	06:07	3.3882	21	46.6110	21.0910	21.0565	0.0345
3	06:12	3.2779	26	46.5430	21.0602	20.9370	0.1232
4	06:16	3.1728	30	46.4690	21.0267	20.8175	0.2092
5	06:20	3.0528	34	46.3160	20.9575	20.6980	0.2595
6	06:24	2.9181	38	46.0490	20.8367	20.5785	0.2582
7	06:27	2.8074	41	45.8360	20.7403	20.4590	0.2813
K	0.4525				$∆d_{max}^{\prime \prime }$	0.1737	

**Table 7:** Resolution estimates of the east–west tilt component at the Chongli Station based on theoretical solid Earth tides.

N	Date	Theory Value	Time Interval	Obs Valus	Norm Value	Fit Value	Difference
	6 November 2025	$d_{i}$	$t_{i}$	$d_{i}^{\prime }$	$d_{i}^{\prime \prime }$	$\bar{d_{i}^{\prime \prime }}$	$∆d_{i}^{\prime \prime }$
	Time	0.001″	min	0.001″	0.001″	0.001″	0.001″
−7	08:21	0.6255	39	−457.4020	−484.0233	−483.8541	0.1692
−6	08:24	0.7444	36	−457.3360	−483.9534	−483.7309	0.2226
−5	08:27	0.8541	33	−457.2280	−483.8392	−483.6077	0.2314
−4	08:31	0.9858	29	−457.1050	−483.7090	−483.4845	0.2245
−3	08:35	1.1009	25	−456.9900	−483.5873	−483.3614	0.2259
−2	08:40	1.2212	20	−456.8740	−483.4646	−483.2382	0.2264
−1	08:46	1.3308	14	−456.7430	−483.3259	−483.1150	0.2109
0	09:00	1.4387	0	−456.4660	−483.0328	−482.9918	0.0410
1	09:15	1.3259	15	−456.5880	−483.1619	−483.1150	0.0469
2	09:21	1.2155	21	−456.5850	−483.1587	−483.2382	0.0795
3	09:26	1.0955	26	−456.6250	−483.2011	−483.3614	0.1603
4	09:30	0.9815	30	−456.6660	−483.2444	−483.4845	0.2401
5	09:34	0.8516	34	−456.7400	−483.3228	−483.6077	0.2850
6	09:37	0.7440	37	−456.8600	−483.4497	−483.7309	0.2811
7	09:40	0.6276	40	−457.0020	−483.6000	−483.8541	0.2541
K	1.0582				$∆d_{max}^{\prime \prime }$	0.1904	

**Table 8:** Comparison of performance parameters: CHP capacitive vs. SQ-70D quartz horizontal pendulum tiltmeter.

	SQ-70D Quartz Horizontal Pendulum Tiltmeter	CHP-Type Capacitive Horizontal Pendulum Tiltmeter
Sensing element	Optical lever	Differential capacitor sensor
Resolution	≤0.0002″	≤0.0002″
Suspension wire	Quartz wire	Tungsten wire
Range	±2″	±3″
Size	Isosceles right-angled triangular base: 47 cm × 47 cm H = 35 cm The optical lever arm length shall be no less than 2 m.	Isosceles right-angled triangular base: 28 cm × 28 cm H = 35 cm Without optical lever
Pendulum locking mechanism	Manually open the cover and lock the pendulum.	Motorized automatic pendulum locking
Scale value	Varies with the i angle Requires monthly calibration	The scale value remains constant over the full measurement range. No calibration is required.
Shock resistance	The pendulum is fragile.	When locked, the instrument can withstand a 1 m drop impact.

## Data Availability

The original contributions presented in this study are included in the article. Further inquiries can be directed to the corresponding author.
